# Diamond Squid (*Thysanoteuthis rhombus*)-Derived Chondroitin Sulfate Stimulates Bone Healing within a Rat Calvarial Defect

**DOI:** 10.3390/md11125024

**Published:** 2013-12-11

**Authors:** Yoshinao Z. Hosaka, Yuji Iwai, Jun-ichi Tamura, Masato Uehara

**Affiliations:** 1 Veterinary Anatomy, Department of Veterinary Science, Faculty of Agriculture, Tottori University, Tottori 680-8553, Japan; E-Mails: hunter.yu.yu.yu@gmail.com (Y.I.); muehara@muses.tottori-u.ac.jp (M.U.); 2 Laboratory of Basic Veterinary Science, United Graduate School of Veterinary Science, Yamaguchi University, Yamaguchi 753-8515, Japan; 3 Department of Regional Environment, Faculty of Regional Sciences, Tottori University, Tottori 680-8551, Japan; E-Mail: jtamura@rs.tottori-u.ac.jp

**Keywords:** bone healing, chondroitin sulfate, diamond squid (*Thysanoteuthis rhombus*), regenerative medicine, BMP-4

## Abstract

Chondroitin sulfate (CS) has been suggested to be involved in bone formation and mineralization processes. A previous study showed that squid-derived CS (sqCS) has osteoblastogenesis ability in cooperation with bone morphogenetic protein (BMP)-4 *in vitro*. However, *in vivo*, osteogenic potential has not been verified. In this study, we created a critical-sized bone defect in the rat calvaria and implanted sqCS-loaded gelatin hydrogel sponges (Gel) into the defect with or without BMP-4 (CS/BMP/Gel and CS/Gel, respectively). At 15 weeks, bone repair rate of CS/Gel-treated defects and CS/BMP/Gel-treated defects were 47.2% and 51.1%, respectively, whereas empty defects and defects with untreated sponges showed significantly less bone ingrowth. The intensity of von Kossa staining of the regenerated bone was less than that of the original one. Mineral apposition rates at 9 to 10 weeks were not significantly different between all treatment groups. Although bone repair was not completed, sqCS stimulated bone regeneration without BMP-4 and without external mesenchymal cells or preosteoblasts. Therefore, sqCS is a promising substance for promotion of osteogenesis.

## 1. Introduction

Bone mineralization events are orchestrated under the control of bone tissue-specific cells and secreted matrix proteins. Pre-osteoblasts, derived from mesenchymal stem cells, proliferate and then differentiate into mature osteoblasts, which initiate the secretion of extracellular matrix proteins and mineralization [1]. It is well known that differentiation of osteoblasts includes these distinct developmental stages with sequential gene expression specific to osteoblasts [2]. Previous studies have shown that non-collagenous proteoglycans (PGs), such as decorin and biglycan, are localized at the starting point of bone mineralization coupled with calcium accumulation [3].

*In vitro* mineralization studies have shown that PGs affect the driving force of apatite crystal induction depending on their concentration [4,5]. Moreover, it has been reported that PG-deficient mice have altered bone-remodeling processes [6,7]. Thus, PGs are thought to play a principal role in regulating bone mineralization. However, it is still not clear whether or not PGs have an influence on osteoblastic cell differentiation, thereby modulating the subsequent biomineralization process.

PGs are a class of glycosylated proteins that have covalently linked sulfated glycosaminoglycans (GAGs). A large part of each GAG chain is constructed with numerous repeating disaccharide units. Decorin and biglycan, a member of the family of small leucine-rich proteoglycans, have chondroitin sulfate (CS) or dermatan sulfate (DS) in the GAG chain [8]. The disaccharide unit of the CS chain is constructed with glucuronic acid (GlcA) and N-acetyl-galactosamine (GalNAc), and that of the DS chain is composed of iduronic acid (IdoA) and GalNAc. These GAGs have some structural variants modified with sulfate groups at different positions. It is known that the disaccharide unit of the CS chain has several structures, CS A, GlcA1–3GalNAc(4*S*); CS C, GlcA1–3GalNAc(6*S*); CS D, GlcA(2*S*)1–3GalNAc(6*S*); CS E, GlcA1–3GalNAc(4*S*,6*S*); and CS H, IdoA1–3GalNAc(4*S*,6*S*) [9]. On the other hand, the DS chain includes so-called CS B, IdoA1–3GalNAc(4*S*). CS A, B, and C have mono-sulfated structures, whereas CS D, E, and H are oversulfated. Naturally occurring CSes are the co-polymers of these disaccharide units. The ratio of the subclass depends on the source of the GAG.

In previous studies, commercialized squid-derived CS (sqCS), which contains CS A, C and E, has been shown to promote osteoblastogenesis in cooperation with bone morphogenetic protein (BMP)-4 [10,11]. Additionally, bovine derived-CS alone or CS and BMP-4 in collagen coated-titanium implant lead to a higher degree of bone formation [12]. We have also revealed that sqCS has an inhibitory ability on adipogenesis by suppressing the expression of several adipocyte differentiation factors. These findings suggest a therapeutic use of CS to control the mesenchymal stem cell differentiation and tissue regeneration.

Focusing on the growth and differentiating factors, BMP-2, -4, and -7 have mainly been used in the research field of bone regeneration due to their ability to accelerate osteogenesis [13,14,15,16,17]. Heparin [18] and heparan sulfate (HS) [19,20] have the ability to bind to fibroblast growth factor (FGF) and are involved in the regulation of osteogenesis. Moreover, heparin has been reported to potentiate the *in vivo* ectopic formation induced by BMP-2 [21]. Although BMPs and HS are one of the effective factors for bone formation, clinical application of BMPs is not only difficult due to cost but also the result of the necessary supra physiological doses that increase the risk of side effects. Recently, a technique for extraction and purification of sqCS from diamond squid (*Thysanoteuthis rhombus*) has been established by Tamura *et al.* [22]. Moreover, sqCS has highly stable properties and can be purified in large quantities at low cost.

In this study, we created a critical bone defect in the rat calvaria and implanted sqCS-loaded gelatin hydrogel sponges into the defect. After a certain period of time, we histologically evaluated the tissues that had formed in the defect and examined the applicability of sqCS as a bone-promoting substance.

## 2. Results

**Figure 1:**
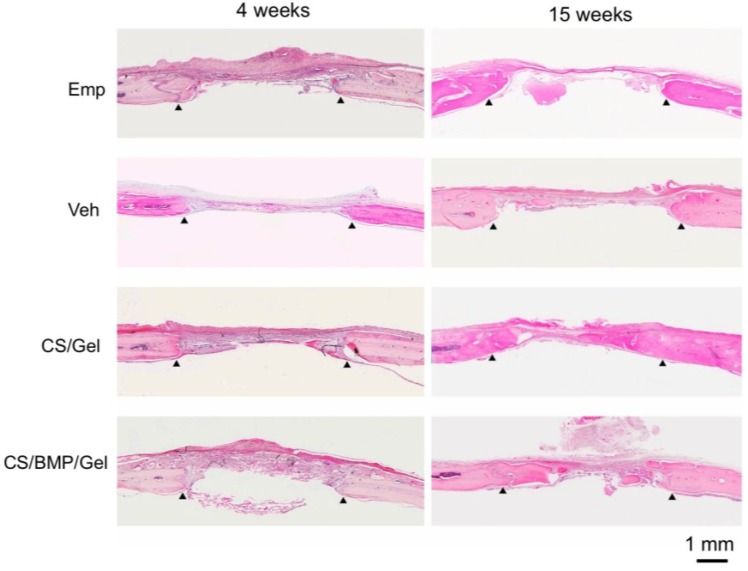
HE staining revealed that bone-like tissues had slightly covered the cut surface of the calvaria in all 4 groups at 4 weeks. At 15 weeks, bone formation was enhanced in the sqCS treated groups (CS/Gel and CS/BMP/Gel) compared to that in the empty defect (Emp) and vehicle (Veh: Sponge only) groups. Arrowheads indicate edges of host bone.

**Figure 2:**
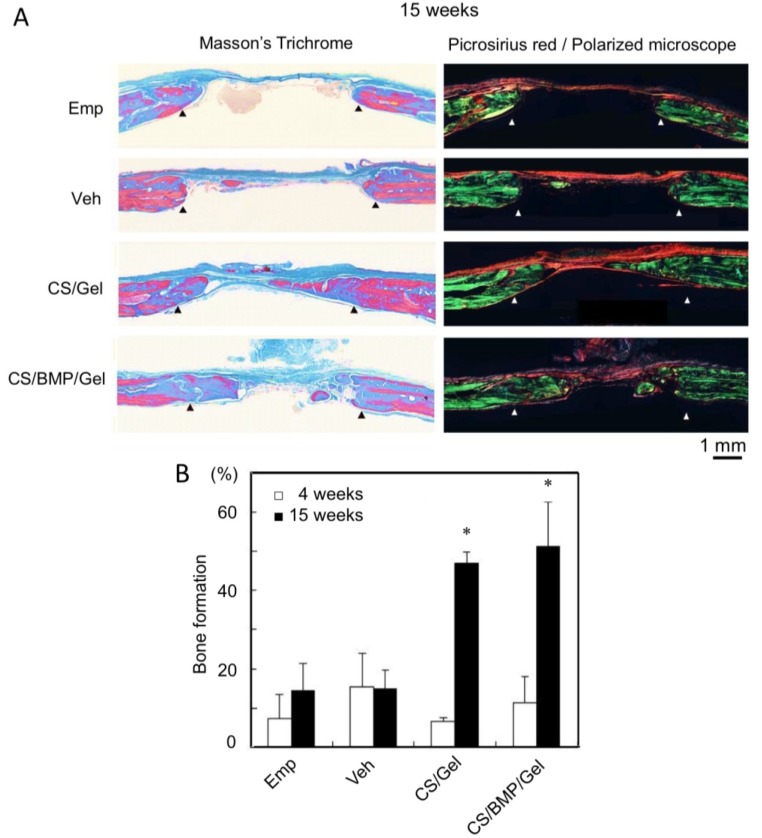
Bone-specific staining of sqCS-treated sponges showed marked bone repair at 15 weeks. (**A**) Masson’s Trichrome (blue: Mineralized-bone, red: Non-mineralized bone) and Picrosirius red/polarized microscopic observation. Arrowheads indicate edges of host bone; (**B**) Percentage of bone formation (%) was determined by image analysis from longitudinal Masson’s Trichrome sections. Significant values are represented as * *P* < 0.05 indicating significant difference from time-matched empty defects.

After calvarial implantation, rats were euthanized at 4 and 15 weeks and defect sites were harvested to evaluate bone repair. The bone-healing area was stained with hematoxylin and eosin (HE) (Figure 1). At 4 weeks, bone-like tissues had slightly covered the cut surface of the calvaria in all 4 groups. The defects in the groups of empty defects and gelatin hydrogel sponge alone (vehicle) remained empty throughout the study. At 15 weeks, CS/Gel and CS/BMP/Gel had stimulated bone repair compared to bone repair in the vehicle and empty defect groups (Figure 1). By observing picrosirius red-stained specimens with a polarizing microscope, it is possible to recognize the colors as density of collagen fibers [23,24]. Applying this principle, we compared the collagen densities of bone and bone-like tissue (new bone), which formed in the defected area of the skull. In the CS/Gel and CS/BMP/Gel groups, new bone showed the same color as that of the original bone tissue (green), and the new bone area was overlapped on a Masson’s Trichrome-stained specimen (Figure 2B). At 4 weeks, neither CS/Gel (6.8% ± 1.1%) nor CS/BMP/Gel (11.4% ± 6.6%) had stimulated bone repair compared to bone repair in the empty defect group (15.5% ± 6.2%) and vehicle group (15.5% ± 8.6%). At 15 weeks, repair rates in the CS/Gel-treated defects and CS/BMP/Gel-treated defects were 47.2% ± 2.8% and 51.1% ± 11.4%, respectively, whereas those in the empty defects and vehicle-treated defects were only 11.0% ± 5.2% and 15.1% ± 4.8%, respectively (Figure 2B). As expected, bone-healing efficacy was in the order of empty defect = vehicle < CS/Gel = CS/BMP/Gel at 15 weeks.

**Figure 3:**
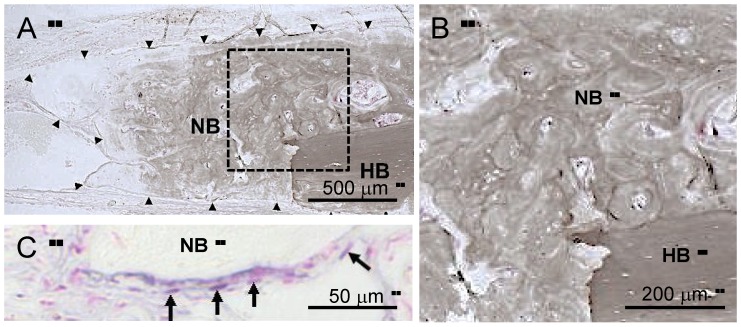
Von Kossa (calcium phosphate-specific staining)-ALP (osteoblast marker) stain of CS/Gel at 15 weeks in undecalfied sections (dark brown: Bone, purple: Osteoblast, pink: Nucleus). (**A**) Level of von Kossa staining of the new bone (NB) was less than that of host bone (HB) and slight mineral apposition was observed at the distal end of the new bone; (**B**) The Harversian system (osteon)-like structure was observed in the NB area; (**C**) ALP-positive cells were observed surrounding the distal end of new bone. Arrowheads surrounding the NB area (**A**) and arrows indicate ALP-positive cells, osteoblasts (**C**). **B** is an enlarged view of the dashed line-square in **A**.

Although the level of von Kossa staining in new bone was less than that in the original bone, the Harversian system (osteon)-like structure was observed in the sqCS-treated calvaria (CS/Gel, CS/BMP/Gel) and slight mineral apposition was observed at the distal end of the new bone (Figure 3A,B). In addition, ALP-positive cells were observed in the periphery of new bone (Figure 3C).

**Figure 4:**
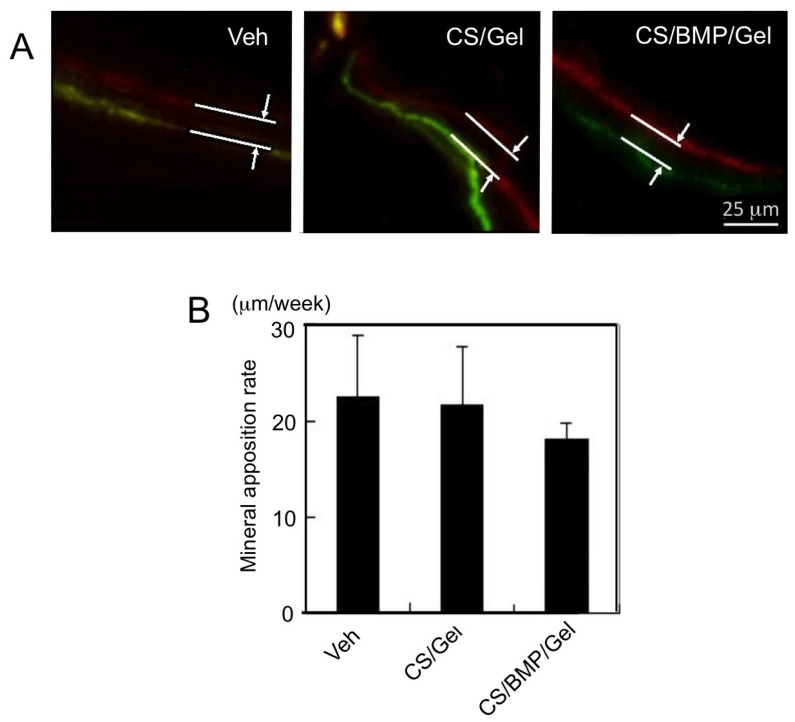
Mineral apposition rates were similar for all treatments. (**A**) Fluorochrome labeling of the mineral front (red: alizarin, green: calcein). Doses were injected 6 and 5 weeks prior to euthanasia (at 9 and 10 weeks), and (**B**) inter-label thickness per dose interval was determined by image analysis to give mineral apposition rate.

In order to evaluate the mineral apposition rate, fluorochrome labels were administered to rats at intervals of 6 to 5 weeks prior to euthanasia at 15 weeks (at 9 and 10 weeks *post* surgery). Clear double labels of calcein and alizarin red S can be seen in Figure 4A, indicating bone formation at 15 weeks *post* surgery. However, at the time points the rate of mineral apposition was equivalent in all groups B). During the measurement, it was noted that fluorochrome labels were observed at a site closer to the periphery of new bone, in the vehicle, CS/Gel and CS/BMP/Gel groups (not shown).

## 3. Discussion

This study provided an approach to evaluate skull regeneration in a rat calvarial defect being stimulated by sqCS-loaded sponges. The histological data clearly demonstrated significant bone repair from sponges treated with sqCS compared to non-treated sponges within 15 weeks *in vivo*.

Miyazaki *et al.* [10] reported that oversulfate CS (type E) bound to BMP-4 and enhanced osteoblast differentiation *in vitro*. Before starting this study, we expected that CS/BMP/Gel would accelerate bone formation more than sqCS alone (CS/Gel) *in vivo*. However, we found no significant difference between the rates of bone regeneration in the CS/Gel and CS/BMP/Gel groups in the present study. It is well known that BMP-4 promotes osteogenesis [25,26]. In a cell culture study, preosteoblasts (MC3T3-E1 cells) continuously expressed BMP-4 [27], suggesting that BMP-4 is capable of stimulating the mineralization of MC3T3-E1 cells [28]. Stadlinger *et al.* examined the effect of bone formation applying CS, recombinant human BMP-4 and collagen on the dental implant surface. In their study, high dose of BMP-4 (2 μg/implant) applied with CS/collagen composite enhanced bone formation [12], but low dose of BMP-4 (400 ng/implant) had a detrimental effect on bone formation [29]. Moreover, at the result of high dose experiment the degree of bone formation was the as same as the CS/Gel (without BMP-4) treatment group. They speculated the reason for that unexpected result of BMP-4 not to enhance bone formation was due to the applied BMP-4 concentration. We also agree with their speculations. Many studies have shown that BMP-4 is distributed in ossification sites *in vivo* [27,30,31,32,33]. If BMP-4 were not preloaded with sqCS (CS/Gel group), intrinsic BMP-4 were released at the implantation site and interacted with sponges and sqCS. Then intrinsic BMP-4 was stored for a certain time in the vicinity of the sponges, which might accelerate osteogenesis. Thus, the level of intrinsic BMP-4 would be sufficient for osteogenesis at the site of a critical defect area and it is unlikely that BMP-4, which was loaded together with sqCS and implanted into the defect, have less effect on osteogenesis. However, we did not determine the BMP-4 concentration at the site of osteogenesis and further study is therefore needed to determine whether BMP-4 has an effect on bone regeneration *in vivo*.

Fluorochrome treatment revealed clear double labels indicating mineral apposition within the defects; however, the rates of bone formation between 9 weeks and 10 weeks in this study were not significantly different. At 4 weeks after surgery, no significant difference was found in the rate of bone regeneration between the experimented groups. In agreement with other findings [15], it is likely that a difference in mineral apposition rate would be evident between 4 and 9 weeks or that bone formation occurs at similar rates but more extensively within the defect site due to sqCS potentiating bone repair throughout the interior of the defect. Interestingly, after 4 weeks surgery, the embedded sponge in the defects of the Empty and Vehicle groups disappeared; however, sponges of CS-loaded groups still remained in the defects. Therefore, both CS-loaded groups might have promoted bone formation by using the remaining sponge. These results also suggest that CS might have a potential to prevent or delay the sponge’s degradation *in vivo*.

Some hypotheses for the role of sulfated GAGs in cell differentiation (osteoblastogenesis) have been proposed. According to the hypothesis, sulfated GAG directly binds to the receptors on the surface of osteoblasts and regulates bone differentiation signal transduction [34]. Heparin and the less sulfated HS facilitate the binding of FGF-2 to FGF receptors (FGFR), thus augmenting osteoblast proliferation and differentiation [35,36,37]. Heparin also bridges Wnt3a and Frizzled (Wnt receptor) by forming a ternary complex similar to the FGF-2-heparin (or HS)-FGFR model and stimulates osteogenesis [18]. According to another hypothesis, sulfated GAG traps some growth factors (such as, BMPs, FGFs, and TGFs) and forms a “sulfated GAG-growth factor complex” in culture medium or ECM and regulates osteoblastogenesis. For example, HS proteoglycans (PG) can bind heparin-binding growth factors (HBGFs) such as BMPs [23] and FGFs with high affinity and then the trapped HBGFs in the medium are released by heparanase and regulate growth and differentiation of osteoblasts [37,38,39]. In agreement with other findings, CS-PGs, such as decorin and biglycan, have the potential to regulate osteogenesis [40,41,42,43]. However, the mechanisms behind the promotion of osteogenesis by CS and CS-PG are still not clear. Further study is needed to clarify the mechanism by which osteogenesis is promoted.

## 4. Experimental Section

All experiments followed the protocols approved by the Ethics Animal Care Committee of Tottori University, Japan (Approval No. 11-T-27), and were conducted compliance with the NIH Guide for the Care and Use of Laboratory Animals [44].

### 4.1. Experimental Design and Surgical Procedure

Twenty-four 10-to-11-week-old male Wistar rats (250–300 g; Shimizu Kagaku, Kyoto, Japan) were housed in a light- and temperature-controlled environment and given food and water. Rats were anaesthetized with a combination of ketamine (75 mg/kg) and xylazine (10 mg/kg), administered intraperitoneally. The dorsal part of the cranium was shaved and aseptically prepared for surgery, and a sagittal incision of approximately 20 mm was made over the scalp of the animal. The periosteum was removed and a full-thickness bone defect (5 mm in diameter) was trephined in the center of each parietal bone using a slow-speed micro grinder (Ustomed Instrumente, Tuttlingen, Germany) without irrigation to heat-damage the host bone on the edges, while cooling the surgical site with flowing cold saline and without damaging the dura (two implants per calvaria). The sqCS containing CS A, C, E and chondroitin (non-sulfated type) in 40, 12, 14 and 34%, respectively, were obtained from the skin of diamond squid (*Thysanoteuthis rhombus*) [12]. Bone defects were randomly implanted with commercialized porcine skin derived-gelatin hydrogel sponges containing beta-tricalcium phosphate (MedGel SP PI9, MedGel, Kyoto, Japan) preloaded with sqCS (CS/Gel; 2 mg/defect) or with sponges preloaded with 2 mg sqCS and 5 μg recombinant human BMP-4 (CS/BMP/Gel; Sigma Aldrich Japan, Tokyo, Japan) or with untreated sponges (vehicle, loaded with PBS only) or left empty. The amount of sqCS and BMP-4, which was loaded on the sponges, was based on the experiment results of Miyazaki *et al.* [10]. The detailed property of the sponges used in the present study has been reported: porcine skin source; 100–300 μm pore size of the sponges; and ethyleneoxide gas sterilization [45]. Just before embedding the sponge into the defects, sqCS (or sqCS and BMP-4) were diluted in 50 μL phosphate buffer saline (pH 7.4), and were dropped on the sponge. The dropped solution containing sqCS and BMP-4 stayed entirely in the sponges. Incisions were sutured and animals were allowed to recover for 4 and 15 weeks, after which they were sacrificed by ether inhalation.

### 4.2. Mineralization Label by Fluorochrome

To label the mineralization front, some of the rats were injected intraperitoneally with calcein (10 mg/kg; Sigma Aldrich, St. Louis, MO, USA) and alizarin red S (20 mg/kg; Sigma Aldrich, St. Louis, MO, USA) six and five weeks prior to euthanasia (nine-week and ten-week time points), respectively.

### 4.3. Histological and Histomorphometrical Analyses

For processing decalcified paraffin sections, parietal bone was fixed in 10% neutral buffered formalin for 72 h at 4 °C and decalcified in Morse’s solution for 72 h at 4 °C with agitation. After dehydration, the calvaria were embedded in paraffin and 8 µm longitudinal sections were used for histological/histomorphometrical (hematoxylin and eosin (HE) stain and Masson’s Trichrome stain) analysis. After scanning the stained sections, bone-healing areas in the specimens were measured. Image-processing software was used to measure the entire area of the defect. From the ratio of these areas, the degree of healing was calculated. Based on the measured areas, the defect repair rate was calculated using the following formula.

Defect repair rate = 100 × (A − B)/A

where A is the ratio of the area of the entire defect on rat calvaria and B is the area of the new bone formation area.

Other decalcified paraffin sections were stained for 1 h in picrosirius solution (0.1% solution of Sirius Red F3BA in saturated aqueous picric acid: Sirius Red F3BA obtained from Sigma Aldrich, St. Louis, MO, USA), and the stained sections were then washed for 2 min in 0.01 N HCl, dehydrated, cleared and mounted. And stained sections were examined under the polarization microscope (S-KE, Nikon, Tokyo, Japan). Some parietal bones were excised and fixed in 10% neutral buffered formalin for 24 h and then in 70% ethanol prior to resin processing/embedding in methylmethacrylate. Sections were cut to 5 μm and some sections stained with von Kossa and alkaline phosphatase (ALP) to identify new bone formation and osteoblasts. Sections were reacted with 5% silver nitrate (Sigma Aldrich, St. Louis, MO, USA) solution in front of 100 W lamps for 1 h, before being rinsed in distilled water and fixed with 5% sodium thiosulfate. Some sections were examined for ALP detection by bromo-chloro-indolyl phosphate/nitro blue tetrazolium chloride with an ALP activity detection kit (Takara, Shiga, Japan). Left sections were unstained for detection of fluorochrome labels and were examined under a fluorescence microscope (IX71, Olympus, Tokyo, Japan); the distances of mineralization fronts (labels of calcein and alizarin red S) were measured, and the growth rate of bone was calculated.

### 4.4. Statistical Analysis

At least three defects per group were used at each time point (4 and 15 weeks) per analysis. Statistical analysis was performed for all of the quantitative results using Student’s *t*-test for comparing means from two independent sample groups. A confidence level of 95% was used (*p* < 0.05).

## 5. Conclusions

This study provided a comprehensively integrated approach to evaluate skull regeneration in a rat critical-sized defect stimulated by sqCS. Histological data clearly demonstrated that significant bone repair occurred in the sqCS-loaded bone defect *in vivo*. Although bone repair was not completed, sqCS stimulated bone regeneration without BMP-4 and without external mesenchymal cells or preosteoblasts. Therefore, sqCS is a promising substance for promotion of osteogenesis.
